# The Effects of Systematic Environmental Manipulation on Gait of Older Adults

**DOI:** 10.3390/healthcare8040386

**Published:** 2020-10-06

**Authors:** Max Toepfer, Alejandra Padilla, Kevin Ponto, Andrea H Mason, Kristen A Pickett

**Affiliations:** 1 Program in Occupational Therapy, University of Wisconsin-Madison, Madison, WI 53750, USA; mtoepfer@wisc.edu; 2 Department of Kinesiology, University of Wisconsin-Madison, Madison, WI 53750, USA; aspadilla@wisc.edu (A.P.); amason@education.wisc.edu (A.H.M.); 3 Department of Design Studies, School of Human Ecology, University of Wisconsin-Madison, Madison, WI 53750, USA; kbponto@wisc.edu; 4 Wisconsin Institute for Discovery, University of Wisconsin-Madison, Madison, WI 53750, USA

**Keywords:** gait, kinematic, falls, older adults, dual task, cognitive load, physical environment

## Abstract

Quantification of gait changes in response to altered environmental stimuli may allow for improved understanding of the mechanisms that influence gait changes and fall occurrence in older adults. This study explored how systematic manipulation of a single dimension of one’s environment affects spatiotemporal gait parameters. A total of 20 older adult participants walked at a self-selected pace in a constructed research hallway featuring a mobile wall, which allowed manipulation of the hallway width between three conditions: 1.14 m, 1.31 m, and 1.48 m. Spatiotemporal data from participants’ walks were captured using an instrumented GAITRite mat. A repeated measures ANOVA revealed older adults spent significantly more time in double support in the narrowest hallway width compared to the widest, but did not significantly alter other spatiotemporal measures. Small-scale manipulations of a single dimension of the environment led to subtle, yet in some cases significant changes in gait, suggesting that small or even imperceptible environmental changes may contribute to altered gait patterns for older adults.

## 1. Introduction

Various environmental features are categorized as regulatory features if they are known to affect performance on motor tasks [1]. This has been demonstrated by Hillel et al. [2] and Bock and Beurskins [3] who found significant differences in the gait of older adults while walking in laboratory and non-laboratory conditions. While we know older adults rely more on environmental cues during the performance of motor tasks than younger adults [4,5], it is possible they are also more sensitive to environmental features. Thus, the salient conditions of one’s environment, such as the size of the walkable area, may be regulatory features that influence spatiotemporal aspects of gait. The resulting changes in gait may mirror more conservative gait patterns similar to those typically observed during lab-based analysis of dual task gait. 

Dual task gait studies allow for tasks with various levels of cognitive demand to be paired with instrumented walking tasks. An inverse relationship has been identified between measures of cognition and fall risk [6,7]. Hindered executive function is specifically associated with gait disruption and increased risk of falls in older adults with [6,8] and without a diagnosis of cognitive impairment [9]. 

As cognitive load increases, specific changes in gait have been observed that correlate to an increased fall risk, mirroring the gait changes observed in older adults with pathology. While walking under a cognitive load, older adults walk significantly slower [10,11,12], with greater variability in their step and stride time [10,13], and greater stride width [11]. These deficits in gait are exacerbated by both an increase in cognitive load [10] and age [14]. A possible explanation, proposed by Yogev-Seligmann et al. [8], for the observed effects of aging on gait is that older adults have fewer attentional resources to devote to different tasks. In other words, older adults are unable to attend to both tasks simultaneously, causing gait to suffer. However, the gait-related effects of the cognitive task seem to be influenced by the nature of the task itself, as arithmetic has been found to affect spatiotemporal parameters of gait in a different manner than verbal fluency tasks [12,15]. While applying a cognitive task is a traditional laboratory method to study gait in a manner that simulates everyday functional tasks, it may not provide context that best fits understanding the sensory and motor aspects of everyday life.

Little research has explored the extent to which altering the environment around a gait task can affect gait parameters. Schaefer et al [11] performed a study comparing the prioritization of gait between older adults and younger adults in a virtual reality setting when completing a cognitive task and walking on a treadmill. Both older adults and younger adults walked significantly slower and with wider step widths when walking in a narrower simulated track compared to a wider track without the presence of an additional cognitive task; however, older adults committed significantly more miss-steps, defined by walking off the virtual track, than younger adults. While the authors did not collect gait variability data, this increase in miss-steps could potentially result in increased gait variability similar to what one would expect during a traditional dual task condition. To add to the body of literature and better understand the effects of the environment on older adults, this study explored whether systematic manipulation of a single dimension of the physical environment can alter spatiotemporal parameters of gait in older adults. We hypothesized that small alterations of the width of a hallway would result in changes to spatiotemporal gait measures and gait variability in a manner similar to what has been previously reported during dual task gait. 

## 2. Materials and Methods 

### 2.1. Participants

Twenty healthy older adults (11 females, mean age 73.58 ± 5.54 years; range: 66 to 88) participated in the experiment. Participants were recruited from the community using flyers. Inclusion criteria included (1) being 65 years of age or older, and (2) being able to walk continuously for 10 min without assistance. Exclusion criteria included (1) any self-reported neurological injury or pathology, or (2) orthopedic conditions limiting function in the last 6 months. Written and informed consent was obtained before starting each session. This study was conducted in agreement with the Declaration of Helsinki and approved by the University of Wisconsin—Madison Social and Behavioral Sciences Institutional Review Board (no. 2018-0865). 

### 2.2. Apparatus

**Figure 1:**
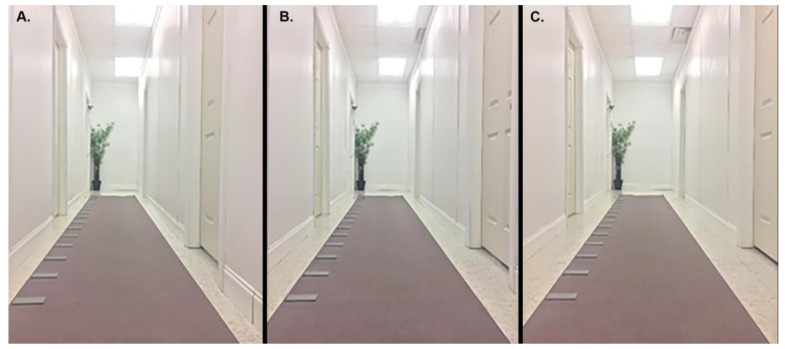
Hallway width conditions. The right wall was positioned in one of three experimental conditions: (**A**) narrow, (**B**) medium, or (**C**) wide, where the distance from the left wall to the right wall was 1.14 m, 1.31 m, or 1.48 m, respectively. The GAITRite mat was repositioned for each condition to maintain a centralized position.

**Figure 2:**
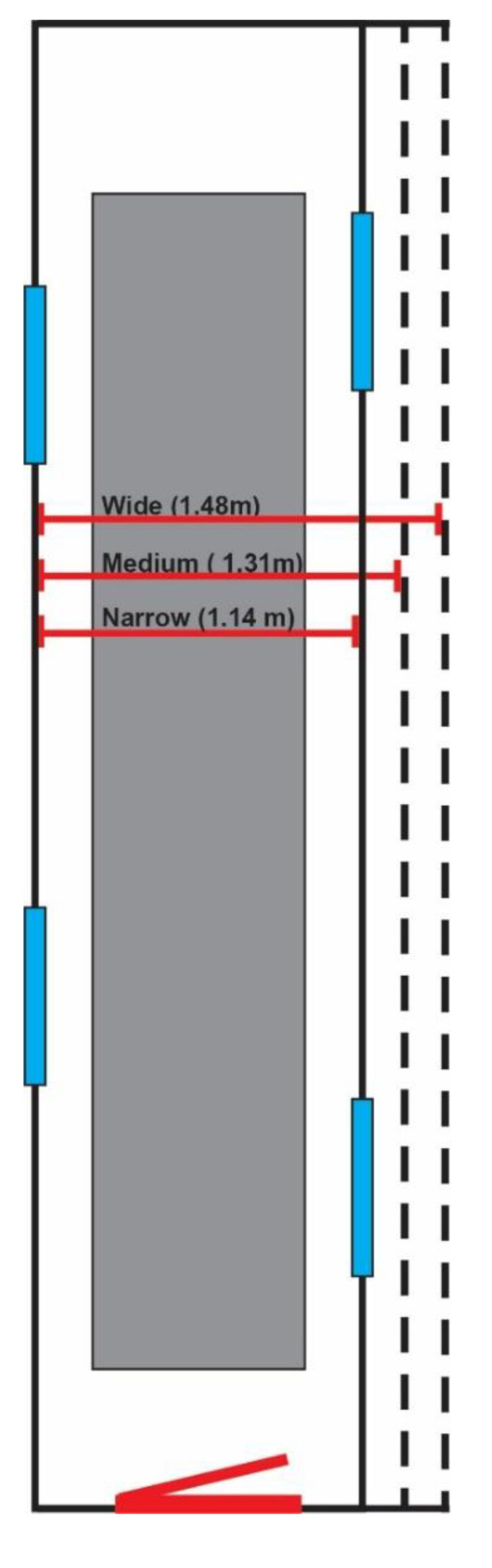
A top-down diagram of the research hallway and the three width conditions. The functional entrance is featured on the bottom of the diagram with the four distractor doors colored in blue.

An 8.13-meter-long hallway was constructed with 1 mobile wall and 1 fixed wall along the long axis of the hallway (Figure 1). The hallway was manually adjusted to 1 of 3 predetermined widths: narrow (1.14 m), medium (1.31 m), or wide (1.48 m) (Figure 2). Hallway width was adjusted by manipulating the mobile wall to the appropriate marked location, which coincided with one of the three predetermined widths. The hallway consisted of a functional door for participants to enter through, and 4 distractor doors, which improved the ecological validity of the scene but were not used by the participant. One distractor door remained ajar to allow for participant cueing to start each trial. A 1.2-meter-tall plant was placed at the end opposite the entrance door to make the scene more realistic.

A GAITRite (CIR Systems Inc., Clifton, NJ, USA) instrumented walkway system was utilized to capture spatiotemporal data. The location of the mat remained consistent along the long axis of the hallway but was manipulated to remain on-center as the width of the hallway was varied. The GAITRite system consists of a 5.12 m long, 0.89 m wide pressure-sensitive mat. At one end of the research hallway, a Logitech (Logitech International S.A., Lausanne, Switzerland) webcam was used to video record participants walking along the mat to reference potential errors in the data resulting from participants stepping off the mat prematurely, or if a participant did not follow protocol. 

### 2.3. Experimental Protocol

Participants’ weight, height, shoulder width, and leg lengths were recorded at the start of each session. Shoulder width was measured using the distance between the maximum bulges of the deltoid muscles as subjects stood against the wall with relaxed arms. Leg length was measured by palpating for the greater trochanter of the femur and measuring to the lateral malleolus of the fibula. 

After the measurements were taken, participants were led to the functional door that opened into the research hallway. A block was initiated when the participant entered the environment, and the door was closed behind them. The first trial began with the individual standing with their back against the functional door. Participants were cued to walk at a comfortable pace across the length of the hallway. Upon reaching the end of the hallway, participants were asked to turn around, face the gait mat, and wait to be cued to start the next trial. Participants followed this protocol for 10 consecutive trials. A block concluded with the participant being cued to leave the research hallway through the functional door. Once out of the research hallway, participants were asked to complete a survey while the hallway width was adjusted to the next condition. Three blocks of 10 trials were completed, for a total of 30 trials. Each block featured a different hallway width: 1.14 m, 1.31 m, or 1.48 m. Participants were not made aware of the hallway width manipulation. Conditions were counterbalanced across participants. Between-block surveys consisted of the Edinburgh Handedness Inventory [16,17] after block 1 and the Waterloo Footedness Questionnaire [18] after block 2. Upon completion of block 3, participants were debriefed and compensated for their time.

### 2.4. Data Processing

GAITRite data were first examined for footfall errors and half steps. Any step that was not completely on the walkway was considered a miss-step. Only trials with at least 4 consecutive valid footfalls were included. Data were then averaged across trials for each condition. Primary outcome measures of step extremity ratio (SER), base of support, percent of gait cycle spent in double support, and velocity were extracted for analysis. SER was calculated by dividing step length by leg length and averaging both left and right ratios. Base of support was defined as the perpendicular distance (cm) between the heel of one foot to the center of the heel of the opposite foot during 2 consecutive footfalls. The percent of the gait cycle spent in double support was defined as the duration of the walk trial the participant spent with both feet in contact with the ground divided by the total time of the walk trial multiplied by 100. Velocity was calculated by dividing the distance between the first and last footfall recorded by the gait mat and dividing by the total time of the trial (cm/s). Finally, the standard deviations of the aforementioned variables were used as metrics of gait variability.

### 2.5. Statistical Analysis

A one-way repeated measures ANOVA using the 3 hallway width conditions as the within-subjects factor was conducted to detect differences in spatiotemporal parameters between conditions. No between-subject factors or covariates were included in this analysis. Significant main effects were further explored via a post-hoc Bonferroni corrected pair-wise comparison. Effect sizes were analyzed using partial eta calculations with small, medium, and large effect sizes defined as 0.0099, 0.0588, and 0.1379, respectively [19]. Data analysis was completed in SPSS v. 26 (IBM, Armonk, NY, USA). 

## 3. Results

Twenty older adults (11 female) completed the study. The average height and weight of the participants were 168.98 ± 8.09 cm and 68.59 ± 12.25 kg, respectively, with mean leg lengths and shoulder breadth equaling 79.85 ± 4.39 cm and 44.33 ± 3.95 cm, respectively. A total of 15 participants were identified as right-handed, 4 as ambidextrous, and 1 as left-handed. Similarly, 16 participants scored in the right-footed range, 3 in the left-footed range, and 1 participant in the neutral range.

Descriptive statistics for participant spatiotemporal parameters across width conditions are included in Table 1. The standard deviation of percent of gait cycle in double support was the only variable to violate sphericity (χ^2^(2) = 28.63, *p* < 0.001). A Greenhouse–Geisser correction was used only for this variable. A repeated measures ANOVA (Table 2) revealed a significant main effect for hallway width on participants’ percent of gait cycle spent in double support (*F* = 5.049, *p* = 0.011) and velocity (*F* = 3.261, *p* = 0.049), both with large effect sizes (*ηp*^2^ = 0.210 and *ηp*^2^ = 0.146, respectively). A post-hoc analysis revealed participants spent significantly more time in double support while walking in the narrow hallway condition compared to the wide condition. Comparisons of the velocity values for the small, medium, and wide hallway conditions did not show significant pairwise differences, likely due to the Bonferroni correction (Table 3). Step extremity ratio and base of support were not significantly different between conditions. Measures of gait variability did not change in response to the hallway manipulation. 

## 4. Discussion

Variations in one’s environment may alter the spatiotemporal characteristics of an individual's gait in a manner similar to a cognitive task. The primary aim of this study was to examine the effect of small alterations of the width of a hallway on spatiotemporal gait measures and gait variability. Our findings indicate that older adult participants spent more time in double support in the narrow hallway walking task than the wide hallway and showed a trend toward slower velocity in the narrow condition. Gait variability was not affected. These findings indicate that subtle environmental changes may influence spatiotemporal gait measures. 

The few studies that have assessed how one’s environment affects gait have often relied on large-scale manipulation in a laboratory setting. In a recent study by Hillel et al. [2], researchers compared the gait of older adults with a history of falling as they walked in a laboratory setting with and without the presence of a cognitive task and compared these findings to measures collected during performance of day to day activities. The results suggest that walking in a laboratory setting may not accurately depict gait in the real world, with step regularity and step time being the least reliable comparisons to walking in the real world [2]. Similarly, Bock and Beurskens [3] conducted a study comparing the gait of older and younger adults in various settings. The authors found that the participants who walked in the laboratory setting walked with smaller limb excursions and greater spatiotemporal variability compared to the group that walked outside in a park [3]. Additionally, the differences in gait parameters between age groups were less pronounced in the group that walked outdoors [3]. In both instances, the large-scale environmental changes allow for the inclusion but not quantification of ecologically relevant variables that are likely contributing to the observed changes.

Instead of comparing gait in two drastically different environments, the present study compared the gait of participants as they walked in conditions differing as little as 0.17 m from the previous width condition. Even so, significant differences were found between the widest hallway condition of 1.48 m and the narrowest condition of 1.14 m. That being said, our results are similar to the findings of Bock and Beurskens [3], as older adults walked with less conservative parameters during the widest hallway width condition, suggesting that the spatial characteristics of the environment can influence gait. By examining subtle manipulations in a functional space, we can better examine the documented adaptations and begin to understand the changes. This study was designed to observe changes in gait after systematic manipulation of the participants’ immediate environment not unlike those observed in real life when transitioning between one hallway and another. While the differences between conditions were minor, effects on gait were observed, which may indicate that the sample of older adults felt less stable and walked more cautiously in the narrow hallway.

During dual task walking, older adults have been shown to walk with reduced speed [12], reduced swing time [20], and significantly greater variability [21]. In this study, velocity and double support were significantly affected by the hallway width. Older adults spent significantly more time in double support while walking in the narrow hallway condition as compared to the wide conditions. Participants also walked slower in the narrow condition, albeit not at a rate significantly different from the medium and wide conditions in the pairwise comparisons. While variability measures did not reach significance, the significant main effect of hallway width on double support and velocity is similar to the findings of Hausdorff et al. [20], who found older adults walking under a dual task condition walked slower and spent less time in swing phase. This decreased time spent in the swing phase results in more time spent with both feet in contact with the ground, similar to the observations from the narrow hallway condition, where participants spent significantly more time in double support. These findings may be relevant to older adult populations at higher risk of falling. Increasing the amount of time spent in double support is a strategy commonly used by individuals who are fearful of falling to limit the amount of time spent balancing on one leg [22]. Findings from the Baltimore Longitudinal Study of Aging [23] and from Springer et al. [24] demonstrate that older adults with a history of falls spend a significantly larger percentage of their gait cycle in the stance phase or double support during dual task walking compared to older adults with no history of falls. If environmental features such as hallway width can alter gait patterns of healthy older adults as our findings suggest, it is possible environmental features carry larger influence on gait than what is currently represented in the literature. 

The current findings are also comparable to those of Schaefer et al. [11], who showed older adults walked with shorter steps and slower velocities, spending more time in double support as the virtual walkway decreased in width. The milder effects observed in the present study could be the result of methodological differences between studies. Unlike Schaefer et al. [11], where participants walked on a self-propelled treadmill and were asked to walk as fast and accurately as possible on a virtual track displayed on a screen in front of them, participants in the present study performed over-ground walking and were not given a task to attend to. Additionally, the emphasis on walking speed could potentially have exacerbated the observed differences. 

Studies of perception and action coupling in standing postures are commonly focused on dynamic changes to the surrounding environment and the resulting postural adaptations [25,26], which seem to become more drastic with age [27]. In the present study, a formal count of which participants noted a change in hallway width during testing was not taken; anecdotally, the majority of participants stated they had not noticed the manipulation during the debrief session. While visual perception of the environment has received a great deal of attention, only one prior study has examined the effects of minor environmental changes on gait. Jetthumrong and Ladavichitkul [28] measured participants’ change in velocity as they walked through a doorway of various widths ranging from 40 to 100 cm. The researchers found inconclusive results as doorway width only affected some individuals within the sample. More work is needed to understand and quantify the extent to which sensory perceptions of the surrounding environment affect gait, particularly for those individuals at a higher risk of sustaining a fall. 

Broad implications cannot be made from the present findings, as the sample is limited to a relatively small size of community-dwelling older adults; however, several implications can be considered for future work. From a clinical and scientific perspective, these findings support the call for standardization of gait assessments, including the environment in which the data are collected. The wide methodological variety found between studies as well as the internal and external factors found to affect one’s gait continues to grow [2], which likely hinders the generalizability of each study’s findings. While these possibly confounding factors may not carry drastic influences on one’s gait parameters, their presence further complicates the ability to accurately tease-apart observed effects imposed by scientific conditions, pathological condition, or improvements following treatment. From a clinical safety and home health standpoint, findings from this study suggest that certain aspects of one’s physical environment, such as narrow rooms and walkways, may influence gait patterns of patients. Further work is needed to explore the perception of environmental factors that influence biomechanical outcomes.

Several limitations of this study must be considered. First, data collection for the present study was suspended prematurely due to concerns of COVID-19, limiting our sample size and strength of observed effects. As a result, we have provided partial eta squared values to aid in interpretation of the findings for the given sample. Additionally, the majority of our sample consisted of fit older adults with no present health concerns or history of falls. Inclusion of a more diverse and representative sample would strengthen the generalizability of the findings. Finally, data regarding whether or not participants recognized the changing widths and their perceptions of each conditions were not formally recorded, and thus conclusions cannot be drawn regarding conscious decisions about motor adaptations and/or anxiety about the testing environment. Future studies should administer more significant environmental changes to observe more pronounced effects and interview the participants after walking in each condition to better understand why these changes occur. 

## 5. Conclusions

The extent to which environmental characteristics affect gait is not well understood. The present study found narrowing a hallway by 34 cm altered the percent of time older adults spent in double support. Future research to quantify environmental influences on spatiotemporal measures of gait are needed to better understand the causes of falls in older adults and work toward fall prevention. 

## Figures and Tables

**Table 1:** Mean spatiotemporal parameters for the three hallway width conditions.

*n* = 20	Narrow (1.14 m)	Medium (1.31 m)	Wide (1.48 m)
Step extremity ratio (SD)	0.802(0.0817)	0.805(0.0793)	0.805(0.08)
Base of support, cm, (SD)	9.451(2.328)	9.214(2.343)	9.305(2.429)
Average percentage double support (SD)	23.999(2.991)	23.798(2.941)	23.515(3.087)
Velocity, cm/s (SD)	122.04(18.086)	123.17(17.945)	123.45(18.019)
Stride length variability (SD)	2.965(0.945)	2.987(0.715)	3.098(0.944)
Base of support variability (SD)	2.077(0.586)	1.924(0.382)	2.007(0.536)
Double support variability (SD) *	0.0159(0.004)	0.019(0.0167)	0.016(0.004)
Velocity variability (SD)	3.978(1.180)	3.869(1.008)	4.172(1.148)

* = Greenhouse–Geisser correction.

**Table 2:** Main effects and effect sizes of the hallway width manipulations on spatiotemporal and variability parameters of gait.

*n* = 20	*F*	*p*-Value	Partial Eta Squared
Step extremity ratio	2.076	0.139	0.099
Base of support	1.668	0.202	0.081
Average percentage double support	5.049	0.011 *	0.210
Velocity, cm/s	3.261	0.049 *	0.146
Stride length variability	0.213	0.809	0.011
Base of support variability	1.051	0.360	0.052
Double support variability	0.978	0.385	0.049
Velocity variability	0.746	0.481	0.038

* = significant (*p* < 0.05).

**Table 3:** Post-hoc pairwise comparison of significant main effects.

Parameter	Hallway Width Condition (a)	Hallway Width Condition Comparison (b)	Mean Difference (a−b)	Standard Error	*p*-Value	95% Confidence Interval for Difference	
						Upper Bound	Lower Bound
Percentage double support	Narrow	Medium	0.202	0.185	0.861	−0.283	0.688
	Narrow	Wide	0.485 *	0.114	0.001 *	0.185	0.785
	Medium	Wide	0.283	0.153	0.240	−0.118	0.683
Velocity	Narrow	Medium	−1.130	0.530	0.139	−2.522	0.262
	Narrow	Wide	−1.410	0.588	0.081	−2.953	0.133
	Medium	Wide	−0.280	0.631	1.000	−1.937	1.377

* = significant (*p* < 0.05).
